# Supplementary material for: End Sequence Analysis Toolkit (ESAT) expands the extractable information from single-cell RNA-seq data
Source: Genome Res. 2016 Oct;26(10):1397–410. doi: 10.1101/gr.207902.116 (PMC5052061; doi:10.1101/gr.207902.116)
Supplement: Supplemental Material [file supp_gr.207902.116_Supp_Materials.tar.gz › ESAT-esat_v0.1/doc/html/enumumms_1_1core_1_1annotation_1_1_annotation_1_1_strand.html]

ESAT: umms.core.annotation.Annotation.Strand Enum Reference


ESAT


- Main Page
- Related Pages
- Packages
- Classes
- Files

- Class List
- Class Index
- Class Hierarchy
- Class Members

All Classes Namespaces Files Functions Variables Enumerator Pages

- umms
- core
- annotation
- Annotation
- Strand

Public Member Functions |
Static Public Member Functions |
Public Attributes |
List of all members

umms.core.annotation.Annotation.Strand Enum Reference

|  |  |
| --- | --- |
| Public Member Functions | |
| String | toString () |
|  | |
| Strand | getReverseStrand () |
|  | |

|  |  |
| --- | --- |
| Static Public Member Functions | |
| static Strand | fromString (String value) |
|  | |

|  |  |
| --- | --- |
| Public Attributes | |
|  | POSITIVE =('+') |
|  | |
|  | NEGATIVE =('-') |
|  | |
|  | UNKNOWN =('\*') |
|  | |

## Detailed Description

Author
:   engreitz Use this enumeration whenever possible to represent strand (rather than using a string or character)

## Member Function Documentation

|  |  |  |  |  |  |
| --- | --- | --- | --- | --- | --- |
| static Strand umms.core.annotation.Annotation.Strand.fromString | ( | String | *value* | ) |  |

static

|  |  |  |  |  |
| --- | --- | --- | --- | --- |
| Strand umms.core.annotation.Annotation.Strand.getReverseStrand | ( |  | ) |  |

Here is the call graph for this function:

|  |  |  |  |  |
| --- | --- | --- | --- | --- |
| String umms.core.annotation.Annotation.Strand.toString | ( |  | ) |  |

## Member Data Documentation

umms.core.annotation.Annotation.Strand.NEGATIVE =('-')

umms.core.annotation.Annotation.Strand.POSITIVE =('+')

umms.core.annotation.Annotation.Strand.UNKNOWN =('\*')

---

The documentation for this enum was generated from the following file:

- java/umms/core/annotation/Annotation.java


---

Generated on Fri Aug 15 2014 15:11:08 for ESAT by  

 1.8.7
